# Global Practice and Efficiency of Multidisciplinary Tumor Boards: Results of an American Society of Clinical Oncology International Survey

**DOI:** 10.1200/JGO.2015.000158

**Published:** 2015-10-28

**Authors:** Nagi S. El Saghir, Raghid N. Charara, Firas Y. Kreidieh, Vanessa Eaton, Kate Litvin, Rania A. Farhat, Katia E. Khoury, Juliana Breidy, Hani Tamim, Toufic A. Eid

**Affiliations:** **Nagi S. El Saghir, Raghid N. Charara, Firas Y. Kreidieh, Rania A. Farhat, Juliana Breidy, Hani Tamim, and Toufic A. Eid, American University of Beirut Medical Center, Beirut, Lebanon; Vanessa Eaton and Kate Litvin,** American Society of Clinical Oncology, Alexandria, VA; and Katia E. Khoury, University Hospitals, Cleveland, OH.

## Abstract

**Purpose:**

Multidisciplinary tumor boards (MDTBs) are universally recommended, but recent literature has challenged their efficiency.

**Methods:**

The American Society of Clinical Oncology (ASCO) conducted a survey of a randomly selected cohort of international ASCO members. The survey was built on SurveyMonkey and was sent via e-mail to a sample of 5,357 members.

**Results:**

In all, 501 ASCO members practicing outside the United States responded, and 86% of them participated in MDTBs at their own institutions. Those who attended represented a variety of disciplines in 70% to 86% of all MDTBs. The majority of MDTBs held weekly specialty and/or general meetings. Eighty-nine percent of 409 respondents attended for advice on treatment decisions. Survey respondents reported changes of 1% to 25% in treatment plans for 44% to 49% of patients with breast cancer and in 47% to 50% of patients with colorectal cancer. They reported 25% to 50% changes in surgery type and/or treatment plans for 14% to 21% of patients with breast cancer and 12% to 18% of patients with colorectal cancer. Of the 430 respondents 96% said overall benefit to patients was worth the time and effort spent at MDTBs, and 96% said that MDTBs have teaching value. Mini tumor boards held with whatever types of specialists were available were considered valid. In all, 94.8% (425 of 448) said that MDTBs should be required in institutions in which patients with cancer are treated.

**Conclusion:**

MDTBs are commonplace worldwide. A majority of respondents attend them to obtain recommendations, and they report changes in patient management. Change occurred more frequently with nonmedical oncologists and with physicians who had less than 15 years in practice. MDTBs helped practitioners make management decisions. Mini tumor boards may improve time efficiency and are favored when the full team is not available. Suggestions for improving MDTBs included making them more efficient, better selection and preparation of cases, choosing an effective team leader, and improving how time is used, but more research is needed on ways to improve the efficiency of MDTBs.

## INTRODUCTION

Management of patients with cancer is becoming increasingly complex and requires a multidisciplinary approach. Multidisciplinary management (MDM) can be accomplished either by multidisciplinary clinics or by multidisciplinary tumor boards (MDTBs).^1^ Breast units are examples of one-stop multidisciplinary clinics in which different specialists see the patient, examine him or her, perform diagnostic procedures such as imaging and biopsies, and make management plans, all in one visit.^2^ An MDTB is defined by the National Cancer Institute as a “treatment planning approach in which several doctors who are experts in different specialties (disciplines) review and discuss the medical condition and treatment options of a patient.”^3^ MDTB meetings usually occur on a weekly basis and involve medical and clinical oncologists, surgeons, radiologists, pathologists, and sometimes specialist nurses.^1^

For decades, MDTBs have been considered the optimal model of care for patients with cancer. MDTBs are generally found in large academic and specialized care centers and are increasingly being established in hospitals and cancer centers worldwide, including in emerging countries.^4,5^ Small, institution-based studies have shown the benefit of MDTBs in patient management.^6–8^ A survey of 338 practicing oncology specialists from various emerging Arab countries showed that 60% of physicians attended MDTBs to seek the opinions of the group and to get help with management plans for their patients.^4^

Given the time and effort spent preparing for and attending MDTBs, their efficiency has recently been the subject of ongoing controversies. Keating et al^9^ recently addressed the issue of MDTB efficiency by surveying 138 Veterans Affairs medical centers in the United States. Surveys asked about the presence of MDTBs at the centers and reviewed a data registry (focusing on data from 2001 to 2004) to study the association between MDTBs and measures of quality cancer care, use of MDTBs, and patient survival. The study did not find a significant effect of MDTBs on patient outcome and challenged their efficiency and worth versus time and effort spent.^9^ After that study, an editorial and a series of letters followed that suggested that MDTBs are still a necessity, especially in rural and low-resource countries in which settings and resources may be suboptimal.^10–15^

We conducted this survey to assess the practice, role, and efficiency of MDTBs to better understand how physicians worldwide use them to make treatment decisions for patients with cancer and to identify areas of strength and weakness to help improve the MDTB tool for better multidisciplinary communication and patient management.

## METHODS

We conducted this MDTB survey on a randomly selected cohort of international members of the American Society of Clinical Oncology (ASCO). The survey was approved by the ASCO International Affairs Committee and was built on the SurveyMonkey Web site. Requests for participation were sent by e-mail in October 2013 to a randomly selected cohort of 5,357 ASCO members living outside the United States, along with two reminder e-mails to the same cohort. Members were advised that ASCO wanted to better understand the use and efficiency of MDTBs in patient care around the world. ASCO members were assured that their answers would be completely anonymous and confidential. Data analysis and multinomial logistic regression were performed by using SPSS (SPSS, Chicago, IL).

## RESULTS

### Characteristics of Respondents

According to ASCO,^16^ the number of ASCO members who practiced outside the Unites States in 2013 was 7,467. Our randomly selected cohort contained 5,357 ASCO members practicing outside the United States of whom 501 (9.35%) responded.

In all, 71.6% of respondents worked at a university teaching or university-affiliated hospital or cancer center, 15% worked at large community hospitals, 1.8% worked at rural hospitals, and 9.7% worked at private clinics. The institutions in which respondents worked had services such as medical oncology (92.1%), pathology (88.1%), surgical oncology (86.5%), radiation oncology (75.9%), diagnostic radiology (75.9%), and palliative care (75.9%).

Of the respondents, 66.9% were medical oncologists, 9.3% were surgical oncologists, and 8% were clinical oncologists (dual medical and radiation oncologists). Their primary area of practice was general oncology (31.1%), breast cancers (19.1%), and GI cancers (11.1%). Regarding years in practice, 34.7% of respondents had been in practice for more than 20 years, 15.6% for 16 to 20 years, 18.9% for 11 to 15 years, 18.4% for 6 to 10 years, and 12.4% for 1 to 5 years in the countries with highest respondents—Canada (8.9%), Australia (8.4%), and Brazil (8.2%).

Of 501 respondents, 451 specified the country of practice. We analyzed demographics according to WHO classification of countries by level of income for the year 2013.^17^ Fifty-nine percent of the 451 respondents were from high-income countries (other than the United States), 25.5% were from upper-middle-income countries, 10.0% were from lower-middle-income countries, and 5.5% were from low-income countries.^17^

### MDTB Attendance

Eighty-six percent of respondents attended MDTBs at their primary institution, whereas 24.6% attended MDTBs at another institution within their city, state, or country. Attendance was multidisciplinary 70% to 86% of the time. Regarding who attended their MDTBs, 86.3% of the medical oncologists said they attended, and 83.7% of surgical oncologists, 80.6% of radiation oncologists, 75.4% of pathologists, 70.8% of radiologists, 60.1% of clinical fellows, 55.3% of residents, and 54% of clinical oncologists said they attended (numbers add up to more than 100% because more than one answer was allowed).

### Reason for Attending MDTBs

Of the 455 ASCO members who responded to the question regarding reason for attending MDTBs, 409 (89%) attended to obtain advice for treatment decisions, 378 (83.1%) to participate in discussions, and 142 (31.2%) said it was mandatory at their institution (numbers add up to more than 100% because more than one answer was allowed).

### Types and Frequency of MDTBs

When asked about the types and frequency of MDTBs, 310 of 456 respondents said they had breast cancer MDTBs at a frequency of once every 1 to 4 weeks, and 240 (77.42%) of these respondents said the MDTB takes place once per week. Among the respondents, 295 said they have GI MDTBs once every 1 to 4 weeks, and 227 (76.95% of these respondents) stated that GI MDTBs occur once per week. A total of 287 respondents said they have thoracic cancer MDTBs once every 1 to 4 weeks, and 204 (78.16% of those) said thoracic MDTBs take place once per week.

### Selection of Patients

**Figure 1:**
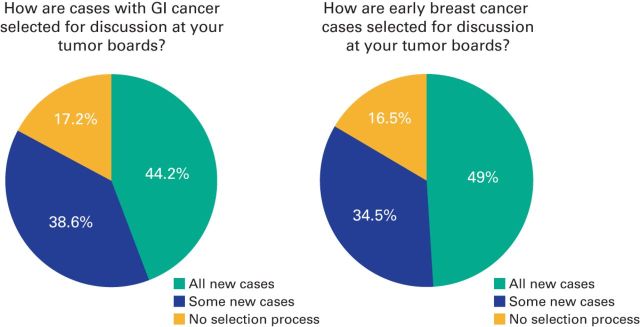
Pie charts showing how patients with GI cancers and early-stage breast cancer are selected for discussion at tumor boards.

Forty-nine percent of respondents reported that all new patients with early-stage breast cancer (Fig 1B) are discussed, 34.5% reported that only some new patients are discussed, and 16.5% had no selection process for patients. Most respondents (48.3%) reported that only a few patients with breast cancer who had disease progression or recurrence were presented, 31.6% of respondents reported that the majority of patients with progression or recurrence were reported, and 20.1% of respondents presented that all of the patients with progression or recurrence were presented. Data showed that 44.2% of respondents presented all patients with newly diagnosed GI cancers at MDTBs (Fig 1A), whereas 38.6% present some new patients, and 17.2% have no selection process. The majority of respondents (49.7%) presented only a few of the patients with CRC who have disease progression or recurrence.

### Documentation of MDTB Recommendations

Notes from the MDTBs are kept in departmental files according to 53.1% of respondents and in the patients' medical records according to 53.6%.

### Changes Made at MDTBs

When asked about how frequently changes were made to the diagnosis and treatment plans for patients with breast cancer or CRC, frequency of changes was divided into five categories: 0%, 1% to 25%, 26% to 50%, 51% to 75%, and 76% to 100%. Diagnosis and treatment plans were divided into the following parameters: change in pathology reading, change in staging, change in the treatment plan as a whole, and change in type of surgery (when applicable). Answers by respondents were transformed into rating averages for comparison.

Of the responders, 410 responded to the question regarding breast cancer and 409 responded to the question regarding CRC. In both breast cancer and CRC MDTBs, the most commonly altered parameter was treatment plan (Table 1). The treatment plan parameter was given the highest average rating of 1.12 for breast cancer and 1.15 for CRC, followed by type of surgery. This implies that physicians relied on MDTB discussions to make final plans for patient management and frequently changed the type of surgery after discussion at MDTB meetings (Table 1).

**Table 1:** Frequency of Changes Made to Diagnosis and Treatment Plans for Patients With Breast Cancer and CRC as a Result of the MDTB Discussion, Based on the Experience of the Respondents

Change Made to Diagnosis or Treatment Plan for Patient	Frequency of Change (%)					Average Rating
	0	1-25	26-50	51-75	76-100	
Pathology changed						
Breast cancer	16.37	46.85	3.53	1.26	0.25	1.01
CRC	24.31	41.10	2.26	1.00	0.50	1.03
Stage changed						
Breast cancer	11.97	48.63	6.23	3.49	0.50	1.03
CRC	10.61	51.01	6.31	2.53	1.01	1.06
Treatment plan changed						
Breast cancer	0.73	44.15	21.71	4.88	2.20	1.12
CRC	1.74	47.89	18.61	2.73	2.73	1.15
Type of surgery changed						
Breast cancer	3.72	49.63	14.64	2.73	0.74	1.04
CRC	3.53	50.63	12.34	2.27	1.26	1.07

Abbreviations: CRC, colorectal cancer; MDTB, multidisciplinary tumor board.

Changes in type of surgery were followed by changes in cancer stage and pathology findings. Respondents ranked change in cancer stage as 1.03 for breast cancer and 1.06 for CRC and ranked change in pathology findings as 1.01 for breast cancer and 1.03 for CRC. This implies that physicians rely heavily on MDTB discussions to finalize diagnoses of their patients and may often make a change in the staging or pathology findings after MDTB discussions (Table 1).

### Multinomial Logistic Regression Analysis of Changes Made at MDTBs

We used multinomial logistic regression analysis to analyze changes at MDTBs according to physicians' specialty and years in practice (Table 2). It showed that, in breast cancer MDTBs, nonmedical oncologists were 2.5 times more likely than medical oncologists to make a change of more than 50% in treatment plans (*P* = .03). In CRC MDTBs, nonmedical oncologists were 3.2 times less likely than medical oncologists to report a change of more than 50% in treatment plan rather than a 0% to 25% change (*P* = .04). Physicians who had been practicing for less than 15 years were 1.21 times more likely in breast cancer MDTBs and 1.75 times more likely in GI MDTBs to make a 50% or more change in treatment plans compared with those with more than 15 years of experience (*P* = .64 and *P* = .28, respectively). For 58.5% of the patients, the main institution required a review of pathology slides from outside hospitals or laboratories.

**Table 2:** Multinomial Logistic Regression Analysis of Changes Made at MDTBs

Type of MDTB	Likelihood of Change in Treatment Plan of More Than 50% in MDTBs According to Specialty or Years of Practice of Presenter		*P*
	Specialty	Likelihood of Change	
Breast tumor	Nonmedical oncologists	2.5 times more likely than medical oncologist	.03
	Physicians with < 15 years of practice	1.21 times more likely than those with > 15 years of practice	.64
CRC tumor	Nonmedical oncologists	3.2 times more likely than medical oncologist	.04
	Physicians with < 15 years of practice	1.75 more likely than those with > 15 years of practice	.28

Abbreviation: MDTB, multidisciplinary tumor board.

### Assessment of the Benefits of MDTBs

Eighty-nine percent of 409 respondents attended MDTBs to seek advice on making treatment decisions, and 83% stated they also attended for the sake of participating in the discussions. Twenty percent of respondents reported that MDTBs always helped them make treatment or diagnostic decisions, 48.1% reported that MDTBs often helped them make those decisions, and only 0.7% reported that MDTBs never helped them make such decisions. When asked whether their participation had helped them make decisions on treatment or diagnostic procedures, a majority of 430 respondents said that MDTBs helped them make management decisions (always: 86 [20%], often: 207 [48%], sometimes: 134 [31%], and never: three [< 1%]).

### Benefits of MDTBs According to Countries' Level of Income

Among respondents from high-income countries, 52.7% stated that MDTBs often helped them make diagnostic and treatment plans, 31.3% stated that MDTBs sometimes helped them do so, and 15.3% stated that MTBDs always helped them. Only 0.8% of those belonging to this category stated that MDTBs never helped them. Among respondents from upper-middle-income countries, 38.6% stated that MDTBs often helped them, 31.7% stated that MDTBs always helped them, and 28.7% stated that MDTBs sometimes helped them. Among respondents from lower-middle-income countries, 45.0% stated that MDTBs often helped them, 30.0% stated that MDTBs sometimes helped them, and 25.0% stated that MDTBs always helped them. Among the respondents from low-income countries, 52.2% stated that MDTBs often helped them, 30.4% stated that MDTBs sometimes helped them, and 17.4% stated that MDTBs always helped them.

### Rating of Suggestions for Improvement

**Figure 2:**
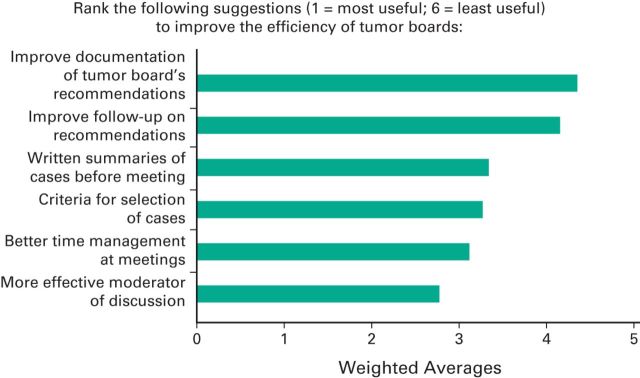
Weighted average of suggestions provided by survey respondents for improving the efficiency of tumor boards. Respondents were asked to rank six suggestions in order of importance, and weighted averages were assigned to each answer choice. Better time management at meetings and more effective moderator of discussions were the most highly ranked suggestions.

Rating averages were used to rank respondents' suggestions on ways to improve the efficiency of MDTBs. Respondents were asked to rank six suggestions in order of importance by using weighted averages assigned to each answer choice. Weighted average is a method of standardization in which a basis of comparison is chosen to provide a standard to which the remaining assigned weighted averages can be compared. The most highly ranked suggestion (Fig 2) was for a more effective moderator of discussions (rating average, 2.77) followed by better time management at meetings (3.12), creating criteria for selecting cases (3.27), and providing attendees with written summaries of the cases before the meetings (3.34). Among the 28 respondents who reported that no MDTB was held at their main institution, 53.6% attributed this deficiency to lack of specialized staff on board, and 89.3% expressed an interest in installing an MDTB.

### Suggestions Made by Respondents

The respondents were asked to list suggestions for improving the efficiency of MDTBs. Among the suggestions were improving infrastructure (bigger rooms, more advanced electronic system for presentation and documentation), employing a clerical person to manage the details, and ensuring that a palliative care consultant was available to participate. Respondents added that MDTB efficiency could be improved by having a coordinator to be responsible for case preparation and to complete presentations. All new cancer cases, related data (eg, names of inpatients and those from clinics, operating rooms scheduled), and the proposed treatment plan would be forwarded to the MDTB coordinator for selection of patients. Patients for whom management is straightforward could be discussed briefly, and patients with complex situations and for whom there seems to be no known guidelines should be discussed in depth. MDTBs should be conducted regularly (weekly), if possible. All discussions should be well documented in patients' medical records, and physicians should provide the MDTB with follow-up for each of the patients discussed.

### Mini Tumor Boards

Mini tumor boards are defined as meetings of a smaller group of specialists who discuss patients and/or treatment plans when there are not enough specialists to represent all areas listed for a comprehensive MDTB. In all, 69.8% of respondents thought that mini tumor boards would reduce time, energy, and effort that might be wasted in a full MDTB; 53.6% were interested in organizing mini tumor boards with available specialists, 31.1% found the idea potentially interesting, and 15.2% were against the idea. The majority of respondents (94.9%) believed that MDTBs (or mini tumor boards) must be made available wherever patients with cancer are treated.

## DISCUSSION

MDTBs are generally a recommended forum for MDM. Summaries of patients' history, physical examination, laboratory results, and imaging and pathology findings are usually presented for group discussions and then plans for management are made. MDTBs provide an opportunity for patients with cancer to get a second opinion through the complementary expertise of a larger number of specialists. Additional plans for diagnosis and treatment are made based on evidence as a best-case scenario. However, because not every clinical patient has evidence-based data, opinions of experts present at the meeting are thought to contribute to collective group decision-making for better patient care.^1^ MDTBs also help improve communication between physicians and may contribute to a better working environment and provide a regular forum for continuing medical education.^18,19^ Although recommendations cover many different areas, efficiency of MDTBs has been the subject of recent debates.^1,9,20–24^

Our survey of a large cohort of international ASCO members showed that MDTBs are commonplace and generally lead to better patient management for physicians from countries with all WHO levels of income. Our survey shows that more than 90% of respondents from high-income countries (outside the United States) and middle- and low-income countries replied that tumor boards are helpful for patient management. The response rate for our survey was only 9.3%, which limits the survey's ability to represent all ASCO members, but the results are still meaningful.

Our study shows that a large majority of respondents (86%) hold MDTBs at their own institutions, with a significant but small percentage who attend MDTBs at nearby institutions. Many respondents lack certain types of specialists at their institutions. Specialty MDTBs are becoming increasingly common worldwide with 81% of respondents reporting availability of general MDTBs, 94% with breast cancer MDTBs, 92% with GI cancer MDTBs, 91% with thoracic cancer MDTBs, and 84.6% with head and neck cancer MDTBs. Our study confirms that multidisciplinary attendance occurs 70% to 86% of the time; however, many respondents still do not have access to MDTBs. Our survey confirms interest in mini tumor boards, which were discussed in an earlier survey of MDTBs in low- and middle-income Arab countries.^4^ Our study shows that about three quarters of respondents hold weekly MDTBs. This leaves approximately 25% with MDTBs once every 2 weeks or monthly, which indicates that there are many patients whose cases are not discussed in MDTBs and thus do not benefit from MDM. Because this survey was limited to ASCO members, the number of patients worldwide who do not benefit from MDM is probably much higher. Efforts to improve the practice of MDM are recommended.

Earlier single-institution studies have shown that patients benefit from changes in diagnosis and treatment plans.^6–8^ The Veterans Affairs study^9^ challenged the benefit as taken for granted and ignited the debate to improve the efficiency of MDTBs. Changes in treatment and benefit for the patient may be smaller at institutions that have several experts if individual physicians always practice evidence-based medicine.^15^ Our survey shows that many respondents use MDTBs to make treatment decisions in the initial planning phase; therefore, changes in diagnosis or treatment plans may not be easy to show. In fact, our survey shows only 1% to 25% change in type of surgery or treatment plan in 44% to 49% of patients with breast cancer and 47% to 50% for patients with CRC; 25% to 50% change in type of surgery or treatment plan was reported for 14% to 21% of patients with breast cancer and for 12% to 18% of patients with CRC. Results of a multinomial logistic regression analysis showed that nonmedical oncologists were 2.5 times more likely than medical oncologists to have a change of more than 50% in treatment plans for breast cancer MDTBs (*P* = .03). Physicians who have been practicing for less than 15 years were 1.21 times more likely in breast cancer MDTBs and 1.75 times more likely in GI MDTBs to have a change of 50% or more in treatment plans compared with physicians who had more than 15 years of experience (*P* = .64 and *P* = .28, respectively). Change of treatment occurs more frequently when patients with breast cancer are presented by nonmedical oncologists.

Our study shows that 89% of 409 respondents attended MDTBs for advice on treatment decisions. This trend is important because it shows increasing reliance on MDTBs worldwide and confirms the need to improve the efficiency of MDTBs. Twenty percent of 430 respondents said that MDTBs help them make management decisions, 96% said that the overall benefit to the patient is worth the time and effort spent at MDTBs, and 96% said that MDTBs have significant teaching value. Suggestions from respondents to improve efficiency emphasized the role of a more effective moderator, better time management at meetings, established criteria for selecting cases, and written summaries before meetings. Respondents favored mini tumor boards held with whichever specialists were available and noted that they may reduce time, energy, and effort spent in full MDTBs; 425 of 448 said that MDTBs should be required for institutions that treat patients with cancer.

In conclusion, our international ASCO survey showed that MDTBs are a useful forum for patient management and for decision making and have become commonplace worldwide. The survey showed that more than 90% of respondents from countries with all WHO levels of income reported that tumor boards were helpful for patient management. Specialty MDTBs are also becoming increasingly common. Change of treatment occurs more frequently when breast cancer cases are presented by nonmedical oncologists and by physicians who have less than 15 years in practice. Survey respondents suggested that MDTB efficiency could be improved by having a coordinator who is responsible for preparing cases, selecting patients, and completing presentations, by having an effective team leader, by using time in a better way, by relying more on existing guidelines, and by using collective expert opinion when necessary. Mini tumor boards may improve efficient use of time and are favored when a full team is not available, especially in low-resource settings. MDTBs provide complementary expertise of a larger number of specialists (a benefit for patients), provide greater likelihood of implementation of evidence-based medicine, and provide expert opinion when evidence is not available. MDTBs may be more helpful in remote areas and in low-resource settings. MDTBs are important and more research is needed to enhance their practice and efficiency. Advances in technology and media are increasing the possibility of reliance on video links and teleconferencing. Such technology can link all levels of peripheral and remote hospitals to academic cancer centers around the world. ASCO has multidisciplinary cancer management courses and courses especially for low- and middle-income countries. In addition to this survey, ASCO is committed to encouraging multidisciplinary cancer management and promoting the use of MDTBs.^25^
